# Hypertensive encephalopathy in untreated congenital hypothryroidism: a case report

**DOI:** 10.1186/1687-9856-2015-S1-P103

**Published:** 2015-04-28

**Authors:** Lilibeth January Garcia, Siok Soan Cua, Ma Cynthia Duñgo, Marissa Salas, Magdalena Lagamayo, Maria Ana Berroya, Sheilah Marie Mirasol

**Affiliations:** 1 Our Lady of Lourdes Hospital, Department of Pediatrics, Sta. Mesa, Manila, Philippines

## 

Hypothyroidism is a well-known cause of poor linear growth in children. In long standing untreated congenital hypothyroidism, short stature, mental retardation and myxedema are common. Sexual precocity associated with bone age advancement can occur, but hypertensive encephalopathy is rare. This case highlights an uncommon finding of hypertensive encephalopathy in a 10 year old Filipino girl who was diagnosed to have hypothyroidism at 18 months old with presenting symptoms of short stature and constipation. She was treated with levothyroxine but with poor compliance. Because of a 2-week history of vomiting, headache, blurring of vision, and increased sleeping time, she was admitted. She was noted to have short stature (height for age Z score -3.62) and obesity (BMI = 26.3, Z score +2.55, WHO) with myxedematous appearance, stubby fingers and toes, and SMR IV. While in the hospital she had seizures, and developed hypertensive encephalopathy. She was noted to have elevated TSH levels (12.5 mIU/L) with normal FT4 and FT3 consistent with subclinical hypothyroidism. Cranial CT scan revealed hypodense foci on the occipital area with cerebral edema. She was given levothyroxine, mannitol and antihypertensive drugs. Her blood pressure was controlled and edema subsided. Prolonged untreated hypothyroidism with elevation of thyroid stimulating hormone leads to mental retardation, short stature, sexual precocity, and hypertension. TSH and gonadotropin hormones have some regions of identical structures in common and may cross react to the same receptors. Prolonged increase in TSH and early rise in gonadotropin have led to ovarian follicles to secrete estrogen, and consequently, breast developent, menarche and skeletal maturation. The hypertension related to hypothyroidism is a result of increased peripheral resistance, changes in renal hemodynamics and hormones. Early recgonition of symptoms and treatment as well as adherenece to levothyroxine therapy is essential in preventing complications of hypothyroidism.

Written informed consent was obtained from the parent of the patient for publication of this abstract and any accompanying images. A copy of the written consent is available for review by the Editor of this journal.

